# COMPARATIVE ANALYSIS OF IMMUNOLOGICAL PROFILES IN WOMEN UNDERGOING CONVENTIONAL AND SINGLE-PORT LAPAROSCOPIC CHOLECYSTECTOMY

**DOI:** 10.1590/0102-6720201600030009

**Published:** 2016

**Authors:** Marisa de Carvalho BORGES, Tharsus Dias TAKEUTI, Guilherme Azevedo TERRA, Betânia Maria RIBEIRO, Virmondes RODRIGUES-JÚNIOR, Eduardo CREMA

**Affiliations:** 1 Department of Surgery, Federal University of Triangulo Mineiro, Uberaba, MG, Brazil; 2 Department of Immunology, Federal University of Triangulo Mineiro, Uberaba, MG, Brazil

**Keywords:** Cholelithiasis, Laparoscopic Cholecystectomy, Cytokines

## Abstract

**Background:**

Surgical trauma triggers an important postoperative stress response characterized by significantly elevated levels of cytokines, an event that can favor the emergence of immune disorders which lead to disturbances in the patient's body defense. The magnitude of postoperative stress is related to the degree of surgical trauma.

**Aim:**

To evaluate the expression of pro-inflammatory (TNF-α, IFN-γ, IL-1β, and IL-17) and anti-inflammatory (IL-4) cytokines in patients submitted to conventional and single-port laparoscopic cholecystectomy before and 24 h after surgery.

**Methods:**

Forty women with symptomatic cholelithiasis, ranging in age from 18 to 70 years, participated in the study. The patients were divided into two groups: 21 submitted to conventional laparoscopic cholecystectomy and 19 to single-port laparoscopic cholecystectomy.

**Results:**

Evaluation of the immune response showed no significant difference in IFN-γ and IL-1β levels between the groups or time points analyzed. With respect to TNF-α and IL-4, serum levels below the detection limit (10 pg/ml) were observed in the two groups and at the time points analyzed. Significantly higher postoperative expression of IL-17A was detected in patients submitted to single-port laparoscopic cholecystectomy when compared to preoperative levels (p=0.0094).

**Conclusions:**

Significant postoperative expression of IL-17 was observed in the group submitted to single-port laparoscopic cholecystectomy when compared to preoperative levels, indicating that surgical stress in this group was higher compared to the conventional laparoscopic cholecystectomy.

## RESUMO

**Racional:**

O trauma cirúrgico induz resposta de estresse pós-operatório significativo, evidenciado pelos níveis elevados de citocinas, podendo favorecer o surgimento de distúrbios imunológicos. A magnitude de estresse está relacionada ao grau do trauma cirúrgico.

**Objetivos:**

Avaliar a expressão das citocinas pró-inflamatórias (TNF-α, IFN-γ, IL-1β, IL-17) e da anti-inflamatória (IL-4) no pré e pós-operatório de pacientes submetidas à colecistectomia laparoscópica por dois métodos: convencional e por portal único.

**Métodos:**

Quarenta mulheres com colecistolitíase sintomática foram operadas, sendo 21 por procedimento laparoscópico convencional e 19 por portal único. As citocinas TNF-α, IFN-γ, IL-1β, IL-4, e IL-17 presentes no plasma foram quantificadas pelo método de ELISA em dois momentos: no pré-operatório e após 24 h da operação.

**Resultados:**

A avaliação da resposta imune não mostrou diferença estatisticamente significante das citocinas IFN-γ e IL-1β na comparação entre os grupos e tempos analisados. Em relação às citocinas TNF-α e IL-4 os níveis séricos estavam abaixo dos níveis de detecção (10 pg/ml) em ambos os grupos e tempos analisados. Observou-se que as operadas por portal único apresentaram expressão significativa da IL-17A do período pré para o pós-operatório (p=0,0094).

**Conclusões:**

A expressão aumentada da IL-17A no pós-operatório do grupo de portal único pode indicar que o estresse cirúrgico foi maior em comparação ao da colecistectomia laparoscópica convencional.

## INTRODUCTION

Cholelithiasis affects mainly women, at a proportion of approximately 2:1. Numerous conditions contribute to the development of gallstones, with the most important being obesity, hypercaloric diet, diabetes, liver cirrhosis, hemolytic disease, physical inactivity, multiple pregnancies, and long-term treatment with sex hormones^13^. 

Laparoscopic cholecystectomy has become the gold standard for surgical treatment of benign gallbladder disease. The advantages of this procedure compared to laparotomy include smaller incisions, reduced surgical trauma, less postoperative pain, faster recovery times, a shorter hospital stay, and faster return to normal activities and work^1^ ^,^ ^7^ ^,^ ^25^ ^,^ ^26^. The use of a single umbilical incision for gallbladder removal was an interesting innovation and single-incision laparoscopic cholecystectomy has gained momentum in the literature since its description by Navarra et al.^21^.

Surgical trauma triggers an important postoperative stress response characterized by significantly elevated levels of cytokines, an event that can favor the emergence of immune disorders which lead to disturbances in the patient's body defense^12^. The magnitude of postoperative stress is related to the degree of surgical trauma. An uncontrolled hyperinflammatory response due to surgical trauma can cause systemic immunosuppression and can contribute to postoperative morbidity and mortality^8^.

The fact that the cholecystectomy technique is the same for both approaches, conventional and single-incision laparoscopy, raised the hypothesis that any difference in the systemic response can be attributed to differences in the size and number of incisions^15^. According to McGregor et al.^20^, the reduction in the total size of the incision seen in single-port laparoscopic cholecystectomy will result in a decrease in the systemic stress response, with a potential reduction in postoperative morbidity.

We found no studies in the literature evaluating the expression proinflammatory (TNF-α, IFN-γ, IL-1β, IL-17A) and anti-inflammatory (IL-4) cytokines before and after conventional and single-port laparoscopic cholecystectomy. Therefore, the objective of the present study was to evaluate differences in the expression of these cytokines in patients submitted to the two surgical techniques.

## METHODS

A prospective, cross-sectional study was conducted at the Department of Digestive Tract Surgery, University Hospital, Federal University of Triângulo Mineiro. Forty women with symptomatic cholelithiasis, ranging in age from 18 to 70 years, participated in the study. The patients were divided into two groups: 21 were submitted to conventional laparoscopic cholecystectomy and 19 to single-port laparoscopic cholecystectomy. The study was approved by the Research Ethics Committee of the university (Permit No. 2503).

The data of each patient were recorded on an assessment form that contained information such as age, anthropometric variables (weight, height, body mass index - BMI), risk factor for cholelithiasis, and duration (minutes) of the surgical procedures.

Women with symptomatic cholelithiasis older than 18 years, who were able to understand the objective of the study and gave informed consent, were included in the study. Criteria for exclusion were pregnancy, BMI>35 kg/m², presence of systemic diseases, use of medications that would interfere with the immune response, a suspicion or confirmation of liver cirrhosis, coagulopathy (platelet count <50,000/µl), antiplatelet therapy (acetylsalicylic acid and clopidogrel), acute pancreatitis, and jaundice.

### Surgical procedure

Anesthesia was standardized as follows: pre-anesthetic medication administered 3 h before surgery consisting of oral diazepam (10 mg), intravenous midazolam (5 mg) and volume expansion with 1,000 ml saline; induction of anesthesia: alfentanil (30 mg/kg), etomidate (0.3 mg/kg) and atracure (0.5 mg/kg); maintenance of anesthesia: continuous infusion of alfentanil (1 mg/kg/min) and isoflurane (0.5-1.5%); decurarization: intravenous atropine (1 mg) and prostigmin (2 mg).

### Conventional laparoscopic cholecystectomy

The patient was placed in horizontal dorsal decubitus on the operating table and received general anesthesia. Perioperative monitoring consisted of cardioscopy, noninvasive blood pressure monitoring, pulse oximetry, and capnography. The pressure of pneumoperitoneum was maintained at 12 mmHg.

Conventional laparoscopic cholecystectomy consisted of the insertion of four trocars, one 10-mm in the supraumbilical region for placement of the optical system and one 5-mm in the right flank for cranial traction of the gallbladder. The other two working trocars were placed in the same line, a 5-mm trocar in the right upper quadrant for removal of the gallbladder and the other 10-mm one in the epigastrium, on the left side of the round ligament, for dissection and hemostasis.

**FIGURE 1:**
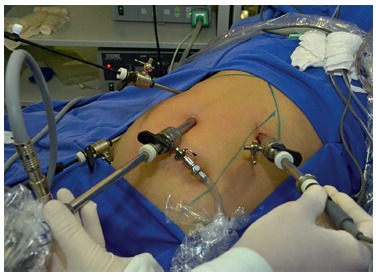
Conventional laparoscopic cholecystectomy.

The hilum was exposed by lateral retraction, holding the infundibulum and retracting it to the right and downwards. The cystic duct was isolated, ligated, and sectioned. Next, the cystic artery was identified, isolated, and clipped with metal clips. The gallbladder was then removed from the liver bed (Figure 1).

### Single-port laparoscopic cholecystectomy

The position of the patients and surgical team, as well as anesthesia and position of the monitor, were similar to those employed in conventional laparoscopic cholecystectomy.A 2-cm transumbilical horizontal incision was made, followed by dissection, opening of the aponeurosis and peritoneum, and placement of a single port (SILS port). Pneumoperitoneum was induced and maintained at 12 mmHg. Trocars were placed through the single port, including two trocars of 5 mm and one of 10 mmHg for introduction of the 30^o^ optic. Conventional laparoscopy materials were used.

After good exposure of the triangle of the bile duct with the aid of a wire passed through the gallbladder infundibulum, dissection, clipping and sectioning of the cystic duct and cystic artery were performed using the same materials as employed in conventional laparoscopy.

**FIGURE 2:**
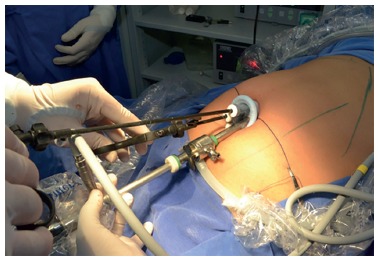
Single-port laparoscopic cholecystectomy.

For clipping, the 5-mm trocar was changed to a 10-mm one introduced in the position. The gallbladder was then dissected from the infundibulum to the fundus (Figure 2).

### Blood collection for quantification of cytokines

Blood samples were collected from all patients after a 12 h overnight fast, before and 24 h after the surgical procedures. The samples were immediately centrifuged at 5,000 rpm and the supernatant was aspirated and stored in 1.5 ml sterile plastic tubes at -70°C.

Plasma levels of TNF-α, IFN-γ, IL-1β and IL-4 (BD Pharmingen, USA) and IL-17 (R&D, USA) were quantified by ELISA. For this purpose, high-affinity 96-well plates (Nunc, Denmark) were sensitized by the addition of 100 µl/well of monoclonal antibodies specific for each cytokine (1 mg/ml in sensitization buffer, pH 9.5) and incubated overnight at 4ºC. Next, the content was discarded and nonspecific binding was blocked by the addition of 200 µl/well of PBS containing 2% bovine serum albumin (Sigma, USA) and incubation for 4 h at room temperature. The PBS-BSA solution was then discarded and samples, diluted 1:2 in PBS 1% BSA, were added to columns 1 to 10 (final volume of 200 µl per well). Serial dilutions (1:2 in PBS-1% BSA, final volume of 100 µl) of the recombinant cytokines were added to columns 11 and 12. The initial concentration was that recommended by the manufacturer for each recombinant cytokine. Wells H11 and H12 were used as blanks and received only 100 µL PBS-1% BSA. The plates were incubated overnight at 4ºC. Next, the plates were washed with PBS containing 0.05% Tween (Sigma, USA) and 80 µl/well of the biotin-conjugated secondary antibody specific for each cytokine was added (1 mg/ml in PBS-1% BSA; all from the same manufacturer as the sensitization antibody). After incubation for 4 h at 37ºC, the plates were washed again with PBS-0.05% Tween, 100 µl peroxidase-conjugated streptavidin was added to each well, and the plates were incubated for 3 h at 37ºC. Finally, the plates were washed again with PBS-0.05% Tween and 100 µl/well of the developing solution containing OPD (Sigma, USA) was added. The plates were incubated at room temperature in the dark. Absorbance was measured at 450 nm in an automated ELISA reader (Bio-Rad 2550 EIA Reader).

The concentration of the cytokines was calculated from a regression line constructed with the absorbances obtained for the recombinant cytokine curve and is expressed as pg/ml. 

### Statistical analysis

Was performed using the Excel 2007 for Windows (Microsoft, USA), Statview (Abaccus, USA) and GraphPad Prism 5.0 (GraphPad Software, USA) programs. The Kolmogorov-Smirnov test was used to determine whether the quantitative variables showed a normal distribution. Continuous variables showing a normal distribution are expressed as the mean±standard deviation and variables that were not normally distributed are expressed as the median and percentiles. Variables showing a normal distribution and homogeneity of variance were compared between the two groups by the Student *t*-test. Variables that showed no normal distribution or homogeneity of variance were analyzed by the Mann-Whitney test for comparison of two independent groups. Repeated measures were evaluated by the Wilcoxon test in the case of two nonparametric samples, by the paired ttest in the case of two parametric samples, and by ANOVA for repeated measures in the case of more than two parametric samples. Differences were considered to be statistically significant when p<0.05.

## RESULTS

Table 1 shows the mean±standard deviation of the age and anthropometric variables of the groups undergoing conventional and single-port laparoscopic cholecystectomy. Height differed significantly between groups (p=0.022). 

**TABLE 1:** Age and anthropometric variables of the groups

Variable	CLC	SPLC	p-value
Age (years)	38,38±11,72	34,21±10,51	NS
Weight (kg)	69,40±16,76	64,81±9,63	NS
Height (m)	1,57±0,07	1,62±0,04	p=0,0220
BMI (kg/m²)	27,90±6,52	24,52±3,67	NS

CLC=conventional laparoscopic cholecystectomy; SPLC=single-port laparoscopic cholecystectomy; BMI= body mass index; NS=not significant. Comparison between genders by Fisher's exact test. Comparison between numerical variables, expressed as the mean±standard deviation, by the Student *t*-test.

CLC=conventional laparoscopic cholecystectomy; SPLC=single-port laparoscopic cholecystectomy.

The mean interval between the beginning and end of the surgical procedures was 62.15±27.75 min in the group undergoing conventional laparoscopic cholecystectomy and 60.12±18.16 min in the group undergoing single-port laparoscopic cholecystectomy. The mean interval between anesthesia induction and the end of the surgical procedures was 83.16±29.20 and 80.50±18.37 min in the groups submitted to conventional and single-port laparoscopic cholecystectomy, respectively.

With respect to risk factor for cholelithiasis questioned in the initial protocol, no significant difference between groups was observed for the following risk factors: physical inactivity, type II diabetes mellitus, high-fat diet, excessive alcohol consumption, use of oral contraceptives, multiple pregnancies, rapid weight loss, obesity, or hereditary factors. However, hyperlipidemia characterized by high blood lipid levels (cholesterol, triglycerides, or both) was significant in the group of patients submitted to conventional laparoscopic cholecystectomy (p=0.0061), as shown in Table 2. 

**TABLE 2:**
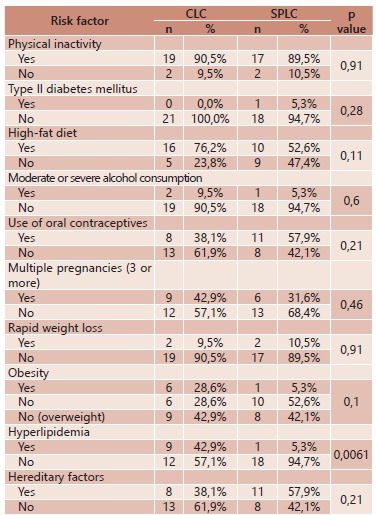
Risk factors for cholelithiasis in patients submitted to conventional and single-port laparoscopic cholecystectomy

None of the following aggravating factors for gallstones was observed in the patients: bile stasis in the gallbladder, hemolytic diseases, biliary infection, primary biliary cirrhosis, and gastrointestinal disorders such as Crohn's disease, ileal resection or bypass and cystic fibrosis with pancreatic insufficiency.

### Immunological evaluation

In the group submitted to conventional laparoscopic cholecystectomy, mean serum IFN-γ levels increased from 336.13±117.31 pg/ml before surgery to 355.93±127.70 pg/ml after surgery. In the group submitted to single-port laparoscopic cholecystectomy, the mean serum levels of this cytokine increased from 472.11±143.17 pg/ml before surgery to 503.42±156.47 pg/ml after surgery. 

**FIGURE 3:**
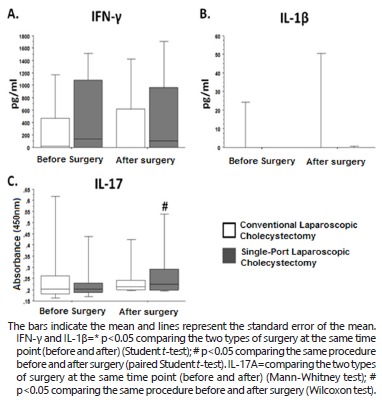
Comparison of IFN-γ (A), IL-1β (B)and IL-17A (C) before and after conventional and single-port laparoscopic cholecystectomy and between conventional vs single-port laparoscopic cholecystectomy at the two time points.

Comparison of mean preoperative serum IFN-γ levels between the groups submitted to conventional and single-port laparoscopic cholecystectomy showed values of 336.13±117.31 vs 472.11±143.17 pg/ml, respectively. Mean postoperative serum IFN-γ levels were 355.93±127.70 in the group submitted to conventional laparoscopic cholecystectomy vs 503.42±156.47 pg/ml in the group submitted to single-port surgery. The serum levels of IFN-γ are shown in Figure 3A.

Mean serum IL-1β levels increased from 12.02±9.42 pg/ml before surgery to 16.40±11.53 pg/ml after surgery in the group submitted to conventional laparoscopic cholecystectomy. In the group submitted to single-port surgery, mean serum IL-1β levels increased from 8.9±8.9 pg/ml before surgery to 9.21±9.13 pg/ml after surgery.

Comparison of mean preoperative serum IL-1β levels between the groups submitted to conventional and single-port laparoscopic cholecystectomy showed values of 12.02±9.42 vs 8.9±8.9 pg/ml, respectively. The mean postoperative serum levels of this cytokine were 16.40±11.53 in the group submitted to conventional laparoscopic cholecystectomy vs 9.21±9.13 pg/ml in the group submitted to single-port surgery. The serum levels of IL-1β are shown in Figure 1B.

Analysis of the expression of IL-17A in the group submitted to conventional laparoscopic cholecystectomy showed mean values of 0.27±0.03 nm before surgery and of 0.26±0.02 nm after surgery. In the group submitted to single-port laparoscopic cholecystectomy, mean IL-17A levels increased from 0.24 ± 0.02 nm before surgery to 0.28 ± 0.03 nm after surgery (p=0.0094).

Comparison of preoperative IL-17A expression between the groups submitted to conventional and single-port laparoscopic cholecystectomy showed values of 0.27±0.03 vs 0.24±0.02 nm, respectively. Mean postoperative expression of this cytokine was 0.26±0.02 nm in the group submitted to conventional laparoscopic cholecystectomy vs 0.28±0.03 nm in the group submitted to single-port surgery. The expression of IL-17 is shown in Figure 1C.

Serum levels of TNF-α and IL-4 were below the detection limit (10 pg/ml) in the two groups and at the time points analyzed.

## DISCUSSION

Cholelithiasis is one of the most common digestive diseases. Women are three times more likely than men to develop gallstones and this gender difference has its onset in puberty and persists throughout the childbearing years^17^.

Age and anthropometric variables such as weight and BMI are intimately related to the presence of gallstones. In the present study, comparison of these variables between the groups submitted to conventional and single-port laparoscopic cholecystectomy showed no significant differences in age (38.38±11.72 vs 34.21±10.51 years) or anthropometric variables such as weight (69.4±16.76 vs 64.81±9.63 kg) and BMI (27.9±6.52 vs 24.52±3.67 kg/m²), although higher values were observed in patients submitted to conventional laparoscopic surgery. In this group, the BMI values confirmed the presence of overweight. There was a significant difference in height (1.57±0.07 vs 1.62±0.04 m) between groups (p=0.022), but this anthropometric variable is not related to the formation of gallstones.

The lipid profile is an important risk factor for the development of gallstones. Low levels of high-density lipoprotein and high serum levels of triglycerides are associated with an increased risk of gallstone formation^11^. In a cross-sectional study involving 75 patients with gallstones, Atamanalp et al.^2^ observed significantly higher serum concentrations of total cholesterol and LDL among patients when compared to the control group. In contrast, a cohort study investigating 2,089 patients reported a strong inverse association between cholelithiasis and plasma cholesterol concentration^23^. In the present study, high serum levels of LDL, total cholesterol and triglycerides, characterizing hyperlipidemia, were observed in nine (42.9%) patients undergoing conventional laparoscopic cholecystectomy and in one (5.3%) patient undergoing single-port surgery (p=0.0061).

With respect to the mean duration of the surgical procedures, a longer operative time has been reported in the literature for single-port laparoscopic cholecystectomy. Sharma et al.^28^ compared the mean duration of surgical procedures and found an operative time of 26 min (15-40 min) for multiport laparoscopic cholecystectomy and of 61 min (40-120 min) for single-port laparoscopic cholecystectomy. In the study of Wagner et al.^33^, the mean operative time was 60 min (33-190 min) for conventional laparoscopic cholecystectomy and 73 min (35-136 min) for single-port laparoscopic cholecystectomy, with a significant difference between procedures (p<0.001)^33^ ^,^ ^29^ ^,^ ^32^. In the present study, no significant difference in the mean interval between the beginning and end of surgery or between anesthesia induction and the end of surgery was observed between conventional and single-port laparoscopic cholecystectomy. However, the mean operative time was shorter for the single-port procedure.

According to Giraldo et al.^26^, women exhibit a greater immune response, both cell-mediated and humoral, are more resistant to infections, and are more susceptible to autoimmune diseases, probably due to the action of female sex hormones. Trastulli et al.^31^ reported that uncontrolled hyperinflammatory responses caused by surgical trauma can lead to systemic immunosuppression, increasing postoperative morbidity and mortality. High circulating levels of cytokines seem to be implicated in the occurrence of complications and in the delay of postoperative recovery of the patient. Attenuation of this presentation would be associated with a reduction in the frequency of complicating factors^28^. Among the responses to disturbances in homeostasis, the acute-phase inflammatory response is important. This response consists of local and systemic reactions aimed at limiting tissue injury, isolating and destroying microorganisms, and activating the repair process necessary to restore the balance of organic functions^14^.

TNF-α and IL-1 are the main mediators of the acute-phase response in humans and are responsible for the activity of extrahepatic manifestations, prostaglandin elevation, tachycardia, and accelerated catabolism^30^. According to Decker et al.^9^, significantly lower serum levels of IL-1 receptors are observed after videolaparoscopy, indicating a lower degree of the inflammatory response to trauma.After surgical procedures and trauma or during infections, TNF-α is one of the earliest and most potent mediators of the inflammatory response. Although the plasma half-life of this cytokine is only 20 min, its expression triggers important metabolic and hemodynamic changes and activates other cytokines distally. TNF-α is a potent inducer of muscle metabolism and cachexia by stimulating lipolysis and inhibiting lipoprotein lipase. Other functions of TNF-α include the activation of coagulation, stimulation of the expression or release of adhesion molecules, PGE2, platelet-activating factor, glucocorticoids and eicosanoids, and its effect on cellular apoptosis^24^.

IFN-γ is produced by three types of cells: CD4^+^ and CD8^+^ Th1 cells and natural killer cells. Together with IL-12 and IL-18, this cytokine plays a fundamental role in the differentiation of CD4^+^ T cells into the Th1 phenotype. IFN-γ also inhibits the differentiation of lymphocytes into Th2 cells. Since Th2 cells secrete the counterregulatory cytokines IL-4 and IL-10, the effect of IFN-γ in reducing the production of these cytokines by Th2 cells further stimulates the development of an inflammatory response against an invading pathogen. IFN-γ also induces the expression or activation of a number of key proteins involved in the innate immune response against microbes^3^.

IL-17 is mainly produced by activated T lymphocytes and stimulates fibroblasts, endothelial cells, macrophages and epithelial cells to produce multiple proinflammatory mediators such as IL-1, IL-6, IL-8 and TNF-α, as well as the activation of NOS2, metalloproteinases and chemokines, inducing inflammation and increasing the expression of intercellular adhesion molecule-1 (ICAM-1)^27^. IL-17A is involved in the protection of the organism against extracellular bacteria and fungi due to its capacity of recruiting neutrophils to the sites of infection. A pathological role of this cytokine has been demonstrated in various models of autoimmune diseases such as experimental autoimmune encephalitis and rheumatoid arthritis^22^.

Comparing the immune responses of patients submitted to laparoscopic vs open cholecystectomy, authors^6^ ^,^ ^19^ observed that the humoral immune responses mediated by IL-4 synthesized by Th2 cells remained unchanged in the laparoscopic and open groups, suggesting that Th2 cell functions and humoral immune responses are only altered by important surgical trauma. In contrast to these findings, Decker et al.^10^ found an increase in IL-4 secretion by T cells after open cholecystectomy.

In the present study, no significant differences in IFN-γ and IL-1β were observed between groups or between the time points analyzed. Comparison of the two surgical techniques showed higher pre- and postoperative serum IFN-γ levels in patients submitted to single-port laparoscopic cholecystectomy, indicating an increase in the inflammatory response in this group. However, we observed a decrease in postoperative serum IFN-γ levels in patients submitted to this surgical procedure, demonstrating that surgical trauma did not trigger an increase in the levels of this cytokine.

Obesity is characterized by the activation of inflammatory processes at metabolically active sites such as the liver, adipose tissue and immune cells^18^. The result of this response is an increase in the circulating levels of proinflammatory cytokines, adipokines and other markers of inflammation. In the present study, one patient (5.3%) with grade I obesity and eight patients (42.1%) with overweight belonged to the group submitted to single-port laparoscopic cholecystectomy. These results may have contributed to the higher postoperative expression of IFN-γ in the group submitted to this surgical procedure.

In the present study, the expression of IL-17A was significantly increased after surgery in patients submitted to single-port laparoscopic cholecystectomy when compared to preoperative levels (p=0.0094). These results show that, although this surgical procedure is associated with less trauma, the anti-inflammatory effects involved in the cellular response to stress were suppressed. The increased postoperative expression of IL-17A in single-port laparoscopic cholecystectomy suggests an important role of this cytokine in tissue repair and in the induction of the inflammatory process.

With respect to TNF-α and IL-4, the serum levels of these cytokines were below the detection limit (10 pg/ml) in both groups and at the time points. 

The inflammatory response to the same type of trauma can vary from patient to patient. Immune-mediated diseases such as rheumatoid arthritis, systemic lupus erythematosus, Crohn's disease, diabetes mellitus and bronchial asthma can alter the serum levels of IL-6. Immunosuppressive or anti-inflammatory drugs such as corticosteroids, which are generally used for the treatment of chronic inflammatory or immune-mediated diseases, modify the cell-mediated and humoral immune response, compromising the serum analysis of inflammatory markers in these patients^5^. In the present study, only one patient (5.3%) of the group submitted to single-port laparoscopic cholecystectomy had diabetes. Furthermore, patients using medications that could interfere with the immune response were not included in the study.

Brune et al.^6^ compared immunosuppression after laparoscopic surgery vs laparotomy. The results showed a significant reduction in IFN-γ (48.3%), TNF-α (36.6%) and IL-2 (36.8%) after laparotomy, but not after laparoscopic surgery. These findings indicated severe suppression of proinflammatory Th1 cytokines after open surgery. In contrast, no significant changes in IL-4 or IL-10 were observed in either group, suggesting that the Th2 cell response and anti-inflammatory activity of these cytokines remained unchanged^6^_._

Berguer et al.^4^ evaluated the production of some intracellular cytokines by T cells after laparoscopic cholecystectomy. The authors concluded that laparoscopic surgery caused mild trauma, but did not activate the production of intracellular IFN-γ by T cells or the IL-4 and IL-10 response during the postoperative period. Han et al.^16^ found the surgical stress response to be the same in conventional and single-port laparoscopic cholecystectomy. McGregor et al.^20^ compared multiport vs single-port laparoscopic cholecystectomy to determine whether a reduced incision size would reduce the response to surgical stress. The authors concluded that no difference exists in postoperative systemic stress, evaluated based on serum levels of IL-6, an important mediator of tissue repair, and C-reactive protein, between the two surgical techniques. However, the group submitted to single-port laparoscopic cholecystectomy showed better recovery characterized by a smaller number of medical intercurrences and fewer surgical wound complications. 

In the present study, no significant differences in IFN-γ and IL-1β were observed between groups or time points analyzed. Comparison between the two surgical techniques showed higher pre- and postoperative serum IFN-γ levels in the single-port group, demonstrating an increased inflammatory response in this group. However, serum IFN-γ levels were reduced after surgery in patients submitted to this procedure, indicating that surgical trauma did not trigger an increase in the levels of this cytokine. 

With respect to IL-1β, comparison between the two techniques showed higher pre- and postoperative serum IL-1β levels in the conventional group, with an increase in the serum levels of this cytokine after surgery compared to the preoperative period. Although these results were not statistically significant, they suggest an increase in chemotactic and phagocytic activities, increased expression of adhesion molecules by endothelial cells, and a consequent increase in clotting activity. 

## CONCLUSION

Significant postoperative expression of IL-17 was observed in the group submitted to single-port laparoscopic cholecystectomy when compared to preoperative levels, indicating that surgical stress in this group was higher compared to the conventional laparoscopic cholecystectomy.
